# From lipid dysbalance to cardiorenal decompensation: apoB/ApoA1 ratio is associated with acute cardiorenal injury in CAD patients

**DOI:** 10.3389/fcvm.2026.1754713

**Published:** 2026-03-13

**Authors:** Liting Pang, Chaoyi Wang, Wenjing Zhao, Lei Cai, Changjie Yu, Sheng Qiu, Qianying Xie

**Affiliations:** Department of Cardiology, Tiantai People's Hospital of Zhejiang Province, Taizhou, China

**Keywords:** ApoB/ApoA1 ratio, apolipoprotein A1, apolipoprotein B, cardiorenal syndrome, coronary artery disease

## Abstract

**Background:**

Cardiovascular and renal diseases exhibit a close bidirectional interaction, which often leads to the development of cardio-renal syndrome (CRS)—a clinical condition in which cardiac dysfunction further aggravates renal injury. Type I CRS is characterized by acute kidney injury secondary to acute heart failure, and this sub-type is closely related to elevated morbidity and mortality in patients with coronary artery disease (CAD). Despite the availability of traditional biomarkers, there is an unmet need for more sensitive indicators to identify high-risk patients for Type I CRS in CAD patients. The apolipoprotein B (ApoB)/apolipoprotein A1 (ApoA1) ratio has emerged as a promising predictor of cardiovascular risk, yet its role in CRS remains unclear.

**Objective:**

This study aimed to evaluate the association between the ApoB/ApoA1 ratio and Type I CRS in patients with CAD, and to assess its value as a biomarker for identifying high-risk patients.

**Methods:**

A retrospective cohort study was carried out on 269 CAD patients complicated with heart failure who were hospitalized in our hospital from 2022 to 2024. According to the estimated glomerular filtration rate (eGFR) results, the enrolled patients were divided into two subgroups: the simple heart failure (SHF) group and the type I CRS group. Data on demographics, clinical history, biochemical measurements, echocardiographic and coronary angiography assessments, and renal function were collected. A multivariable logistic regression model was used to assess the association between the ApoB/ApoA1 ratio and CRS, adjusting for potential confounders. Correlation analyses were performed to explore the relationships between key variables and the occurrence of type I CRS. A multivariable logistic regression model was used to assess the association between the ApoB/ApoA1 ratio and CRS. Furthermore, a receiver operating characteristic (ROC) curve was constructed to evaluate the predictive accuracy of the ApoB/ApoA1 ratio for type I CRS.

**Results:**

A total of 269 patients were enrolled. Significant differences were observed between the simple heart failure (SHF) group and the CRS group in terms of age, history of diabetes mellitus, levels of triglycerides (TG), apolipoprotein A1 (apo-A1), apolipoprotein B (apo-B), ApoB/ApoA1 ratio, and serum creatinine (Scr). Patients in the CRS group were older, had a higher proportion of diabetes mellitus, higher levels of TG, apo-B, and Scr, a higher ApoB/ApoA1 ratio, but lower levels of apo-A1 compared to the SHF group. Multivariable logistic regression analysis identified age and the ApoB/ApoA1 ratio as independent risk factors for CRS. The receiver operating characteristic (ROC) curve analysis showed that the ApoB/ApoA1 ratio had a moderate level of predictive accuracy for Type I CRS, with an area under the curve (AUC) of 0.782.

**Conclusion:**

The ApoB/ApoA1 ratio is moderately associated with the risk of developing Type I CRS in patients with CAD. This ratio could serve as a clinically relevant biomarker for early identification of in-hospital Type I CRS risk in CAD patients with acute heart failure, potentially aiding in the implementation of early and targeted interventions to improve patient outcomes.

## 1 Introduction

Cardiovascular diseases (CVDs) and renal disorders share a complex and bidirectional interplay, often leading to the development of cardiorenal syndrome (CRS), a clinical condition where dysfunction in one organ exacerbates the dysfunction of the other (1, 2). Cardiorenal syndrome (CRS) is divided into five clinical subtypes according to the onset speed and pathological mechanism of organ dysfunction, among which Type I CRS is an acute clinical phenotype—acute heart failure caused by acute coronary events triggers acute kidney injury, and this subtype is the most common in CAD patients with acute decompensation. It is associated with significant morbidity and mortality in patients with coronary artery disease (CAD), highlighting the need for early identification and targeted interventions (3, 4).

CAD, a prevalent form of CVD, is frequently complicated by CRS, which significantly worsens patient prognosis. The incidence of type I CRS in CAD patients is substantial, and it is linked to adverse outcomes, including increased cardiovascular events and mortality (3). Traditional biomarkers such as B-type natriuretic peptide (BNP) and serum creatinine have been used to assess cardiac and renal function, respectively (5, 6). However, there is a critical need for more specific and sensitive biomarkers to identify the risk of type I CRS in CAD patients.

Recent research has focused on lipid-related biomarkers due to their potential role in both atherosclerosis and renal dysfunction. The apolipoprotein B (ApoB)/apolipoprotein A1 (ApoA1) ratio has emerged as a promising biomarker associated with cardiovascular risk. ApoB is a key component of atherogenic lipoproteins, while ApoA1 is the primary protein component of high-density lipoprotein (HDL), which has anti-inflammatory and anti-atherosclerotic properties. The ApoB/ApoA1 ratio is a key index reflecting the balance between pro-atherosclerotic and anti-atherosclerotic lipid components in the body. Existing studies have confirmed its predictive value for cardiovascular adverse events, but its specific correlation with the occurrence of Type I CRS and its underlying mechanism in CAD patients have not been clearly clarified, which is the key research gap addressed in this study (7, 8).

This study retrospectively analyzes the clinical data of CAD patients with acute heart failure, aiming to explore the correlation between the admission ApoB/ApoA1 ratio and the occurrence of Type I CRS within 7 days of admission. We hypothesize that the elevated ApoB/ApoA1 ratio is an independent risk factor for Type I CRS in CAD patients, which has predictive value beyond traditional clinical risk factors. By exploring this relationship, we seek to provide new insights into the pathophysiological mechanisms underlying type I CRS and to identify a potential biomarker for early risk stratification and clinical management.

## 2 Methods

### 2.1 Study design and patient selection

This single-center retrospective clinical study was conducted in Tiantai County People's Hospital, and the research objects were CAD patients hospitalized for acute heart failure from January 2022 to December 2024. CAD was defined as the presence of atherosclerotic lesions in one or more major coronary arteries, confirmed by coronary angiography, with a luminal stenosis of ≥50%. Patients aged 18 years or older were considered for inclusion.

The inclusion criteria were as follows: (1) a confirmed diagnosis of CAD based on coronary angiography; (2) admission primarily due to heart failure, defined by typical symptoms (e.g., dyspnea, fatigue) and signs (e.g., pulmonary congestion, peripheral edema) along with elevated levels of B-type natriuretic peptide (BNP); (3) availability of complete medical records, including laboratory test results and treatment details.

The exclusion criteria were as follows: (1) a history of chronic kidney disease (CKD) defined as kidney damage or a decreased eGFR (<60 mL/min/1.73 m^2^) for ≥3 months; (2) severe liver dysfunction (e.g., liver cirrhosis, acute liver failure); (3) presence of malignant tumors; (4) active autoimmune diseases (e.g., systemic lupus erythematosus, rheumatoid arthritis); (5) severe infections or inflammatory diseases that could affect renal function and lipid metabolism.

Patients aged ≥18 years were eligible for inclusion. Given that elderly patients (≥75 years) commonly exhibit age-related decline in eGFR, we specifically retained this population to reflect real-world clinical practice. Age was treated as a continuous variable in multi-variable analysis to account for its physiological impact on renal function. The proportion of elderly patients and sensitivity analyses are reported in the Results section.

The study strictly enrolled patients with Type I CRS, defined as acute heart failure (triggered by CAD) leading to AKI (eGFR < 60 mL/min/1.73 m²) within 7 days of admission, without pre-existing CKD (excluded via criterion 1). Patients meeting other CRS subtypes were excluded to ensure the study population's specificity.

The study was approved by the Ethics Committee of Tiantai County People's Hospital (Approval No.: 202601081907000099513). In line with the Declaration of Helsinki, individual informed consent was waived due to the retrospective design.

### 2.2 Data collection

Comprehensive data were collected for each patient from electronic medical records and clinical documentation. Demographic information included age, sex, body mass index (BMI), and smoking status. Detailed medical history was recorded, including history of hypertension, diabetes mellitus, hyperlipidemia, previous myocardial infarction, and family history of cardiovascular disease.

Blood samples were collected from each patient after an overnight fast to measure various biochemical parameters. Fasting venous blood samples were collected from all patients at admission, and serum lipid indexes including triglycerides (TG) and total cholesterol (TC) were detected by Beckman Coulter AU5800 automatic biochemical analyzer in accordance with the hospital's clinical laboratory standard operating procedures (9, 10). Assays were also performed to determine the levels of lipoprotein(a) [Lp(a)], apolipoprotein A1 (ApoA1), and apolipoprotein B (ApoB) in serum (11). Meanwhile, serum creatinine and brain natriuretic peptide (BNP) concentrations were quantified to respectively assess renal functional status and cardiac stress response.

All laboratory analyses were performed in the hospital's clinical laboratory, which adheres to standardized operating procedures and quality control measures to ensure the accuracy and reliability of test results.

### 2.3 Echocardiographic assessment

Echocardiographic evaluation was systematically conducted in all study participants using a contemporary Philips EPIQ-7 ultrasound platform. This non-invasive diagnostic technique provided detailed assessments of cardiac structure and function. Measurements included the left atrial diameter (LA), interventricular septal end-diastolic thickness (IVSd), left ventricular end-diastolic dimension (LVEDD), and left ventricular end-systolic dimension (LVESD), all performed in accordance with guidelines established by the American Society of Echocardiography. Additionally, the left ventricular ejection fraction (LVEF), a critical marker of systolic cardiac function, was determined utilizing the modified Simpson's biplane method. This comprehensive echocardiographic data set is integral to understanding the cardiac implications in patients with coronary artery disease and cardiorenal syndrome (12, 13).

### 2.4 Coronary angiography assessment

Coronary angiography was conducted in a specialized catheterization laboratory equipped with advanced imaging systems, specifically utilizing the Allura Xper FD20 by Philips. A minimum of two highly experienced interventional cardiologists independently interpreted the angiograms, meticulously evaluating the severity and nature of the lesions present. The left coronary artery was visualized across five to six different projections, while the right coronary artery was examined in two to three distinct views, ensuring a comprehensive evaluation of the coronary vasculature (14–16). This thorough angiographic assessment is crucial for accurately determining the extent of coronary artery disease, which is essential for guiding subsequent clinical management strategies and interventional decisions.

### 2.5 Assessment of renal function

Renal function was evaluated by determining the estimated glomerular filtration rate (eGFR) using the Chronic Kidney Disease Epidemiology Collaboration (CKD-EPI) equation, which is widely accepted for its accuracy in estimating renal function across different demographics. The equation is given by (17, 18):

$$eGFR=141\times \min(kScr,1{)}^{-0.329}\times \max(kScr,1{)}^{-1.209}\times 0.993\times \mathrm{Ag}e^{-0.233}\times 1.018\times \mathrm{Sex}\times 1.159\times \mathrm{Race}$$

where: *k* is a constant that varies by sex and ethnicity (0.7 for females and 0.9 for males), Sex is 0 if female and 1 if male, Race is 0 if white and 1 if black)

Patients were stratified into two groups based on their eGFR values: those with eGFR less than 60 mL/min/1.73 m² were classified into the cardiorenal syndrome (CRS) group, and those with eGFR equal to or greater than 60 mL/min/1.73 m² were placed in the simple heart failure group (SHF group). This categorization is essential for understanding the renal status of patients and for guiding clinical management and treatment strategies.

### 2.6 Statistical analysis

Statistical computations were all completed using the IBM SPSS Statistics software (Version 21.0). For continuous variables, the mean ± standard deviation (SD) was used for description, and one-way ANOVA was adopted as the parametric test method, with LSD *post hoc* test conducted for further intergroup comparisons. Non-parametric data were depicted as median (interquartile range) and compared by the Kruskal–Wallis test. Categorical variables were summarized as *n* (%) and analyzed with chi-square test or Fisher's exact test in accordance with relevant statistical criteria. Multivariable logistic regression analysis was carried out to assess the association between ApoB/ApoA1 ratio and CRS development, with potential confounding variables incorporated as covariates for adjustment. A two-tailed *P* value of less than 0.05 was regarded as indicative of a statistically significant difference.

Sample Size Calculation: Based on the primary objective of evaluating the ApoB/ApoA1 ratio's identify the risk for Type I CRS, we estimated the sample size using logistic regression formula. Referring to previous literature, the incidence of Type I CRS in CAD patients with heart failure is 40%–60%. Assuming a moderate effect size (OR = 1.5), type I error of 0.05, power of 80%, and adjustment for up to 5 covariates, a minimum of 240 patients was required. Our final sample of 269 patients ensured adequate statistical power. Additionally, the ratio of CRS cases (*n* = 159) to predictor variables (6) was 26.5:1, exceeding the recommended 10:1 for logistic regression model stability.

Additionally, minor missing data was present in 3 key indicators [TG: 1 case, Lp(a): 2 cases, LA: 1 case] with an overall missing rate of 0.7%. Given the random nature of missingness, multiple imputation (MI) with 5 imputed datasets was applied to handle missing values. The imputation model incorporated covariates including age, diabetes mellitus, and serum creatinine to minimize bias. Statistical analyses were performed on the combined imputed datasets, and consistency between imputed and observed data distributions was verified to ensure reliability.

## 3 Results

### 3.1 Patient selection and flow

**Figure 1:**
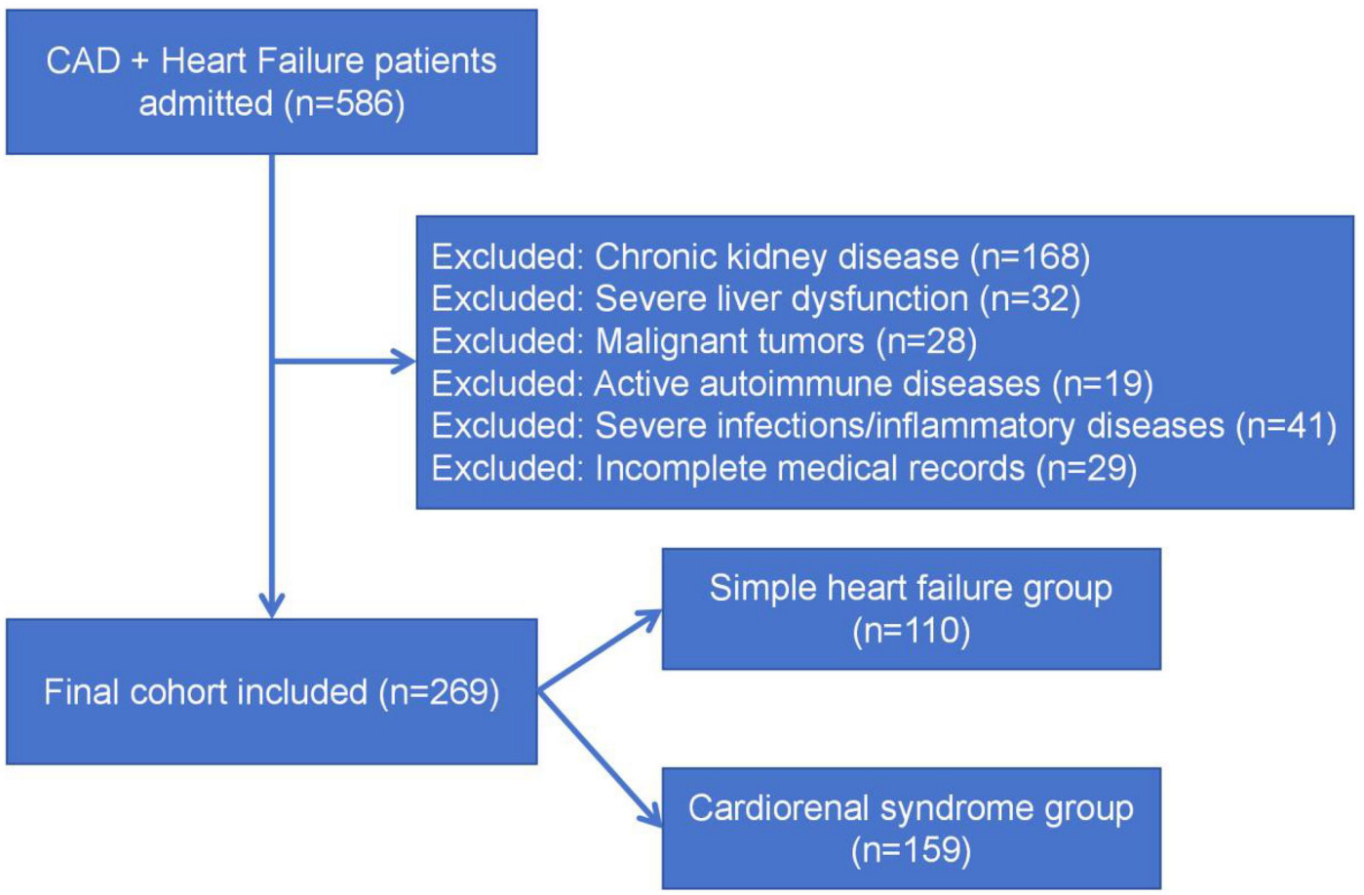
Patient selection flowchart.

During the study period from January 2022 to December 2024, a total of 586 patients with CAD and heart failure were admitted to TianTai Country People's Hospital. After applying the exclusion criteria sequentially, 317 patients were excluded, leaving 269 patients for the final analysis. The detailed screening process is shown in Figure 1.

### 3.2 Among the total 269 enrolled patients, key indicators exhibited reliable distribution profiles

Demographic (age, BMI), core biochemical (apo-B, apo-A1, ApoB/ApoA1 ratio, Scr), and echocardiographic (LVEF, LA, LVEDD) parameters were normally distributed with no extreme outliers. Triglycerides (TG) showed a mild skewed distribution [median (IQR): 1.18 (0.75, 1.32) mmol/L], which met statistical test requirements after appropriate processing. As detailed in Table 1, age, diabetes mellitus, TG, apo-A1, apo-B, ApoB/ApoA1 ratio, and Scr differed significantly between the SHF and CRS groups (all *P* < 0.05), while other indicators showed no statistical differences (all *P* > 0.05).

**Table 1:** Comparison of clinical characteristics of patients.

Item	Simple heart failure group(SHF) (*n* = 110)	cardiorenal syndrome group (CRS) (*n* = 159)	*P Value*
Demographic Characteristics			
Gender (Male, %)	58 (52.7%)	91 (57.2%)	0.226
Age (years)	68.5 ± 12.2	75.7 ± 9.2	0.026
BMI (kg/m^2^)	23.9 ± 3.2	23.6 ± 2.7	0.122
Underlying Diseases			
Hypertension (*n*, %)	61 (55.5)	94 (59.1)	0.114
Diabetes Mellitus (*n*, %)	32 (29.1)	67 (42.1)	0.006
History of myocardial infarction (*n*, %)	14 (12.7)	35 (22.0)	0.052
Biochemical Indicators			
TG (mmol/L)	0.94 (0.63, 1.05)	1.36 (0.82, 1.58)	0.026
TC (mmol/L)	4.23 ± 1.17	4.36 ± 1.06	0.075
HDL-C (mmol/L)	1.17 ± 0.40	1.25 ± 0.37	0.093
LDL-C (mmol/L)	2.34 (1.78, 2.80)	2.41 (1.79, 2.97)	0.077
apo-A1 (mmol/L)	1.22 (0.94, 1.36)	1.06 (0.79, 1.13)	0.000
apo-B (mmol/L)	0. 69 (0. 51,0. 97)	0. 76 (0. 57,1. 04)	0.003
ApoB/ApoA1 Ratio	0. 62 ± 0.19	0. 75 ± 0.21	0.000
Scr (μmol/L)	67.9 ± 19.8	103.0 ± 21.5	0.000
BNP (ng/mL)	573.8 (371.4, 944.6)	652.7 (355.9, 1,052.7)	0.226
Echocardiographic			
LVEF (%)	47.1 ± 15.0	49.1 ± 12.5	0.219
LA (mm)	40.0 ± 9.6	40.1 ± 9.5	0.917
IVSd (mm)	10.4 ± 1.75	10.4 ± 2.1	0.212
LVESD (mm)	36.5 ± 8.3	34.3 ± 10.3	0.448
LVEED (mm)	52.7 ± 7.8	51.9 ± 7.3	0.173

TG, triglycerides; TC, total cholesterol; HDL-C, high-density lipoprotein cholesterol; LDL-C, low-density lipoprotein cholesterol; apo-A1, apolipoprotein A-I; apo-B, apolipoprotein B; Scr, serum creatinine; BNP, B-type natriuretic peptide.

Among the total cohort, 142 patients (52.8%) were aged ≥75 years, with 45 (40.9%) in the SHF group and 97 (61.0%) in the CRS group (*P* < 0.001). To ensure our findings were not driven solely by this elderly subgroup, we performed a sensitivity analysis excluding patients ≥75 years, which confirmed that the ApoB/ApoA1 ratio remained an independent predictor of CRS (OR = 1.423, 95% CI: 1.198–1.815, *P* = 0.032).

### 3.3 Medication use comparison

Table 2 illustrates the comparison of medication use between the SHF group and the CRS group. In terms of antiplatelet drugs, the usage rates of aspirin, indobufen, clopidogrel, and ticagrelor showed no significant differences between the two groups (all *P* > 0.05). For beta-blockers, the differences in the usage of metoprolol and bisoprolol between the groups were not statistically significant (both *P* > 0.05). In the category of lipid-lowering drugs, the use of statins and fibrates did not differ significantly between the SHF and CRS groups (*P* = 0.294 and *P* = 0.115, respectively). Regarding RAAS inhibitors, the usage of ACEIs, ARBs, and ARNIs also showed no significant differences between the two groups (all *P* > 0.05). Similarly, the use of diuretics and MRAs did not present any statistically significant differences between the SHF group and the CRS group (*P* = 0.120 and *P* = 0.072, respectively).

**Table 2:** Comparison of medication use of patients.

Item	Simple heart failure group (SHF) (*n* = 110)	cardiorenal syndrome group (CRS) (*n* = 159)	*P Value*
Antiplatelet drugs			
Aspirin	92 (83.6)	125 (78.6)	0.165
Indobufen	9 (8.18)	20 (12.6)	0.063
Clopidogrel	12 (10.9)	18 (11.3)	0.528
Ticagrelor	17 (15.5)	19 (11.9)	0.169
Beta-blockers			
Metoprolol	47 (42.7)	55 (34.6)	0.102
Bisoprolol	24 (21.8)	47 (29.6)	0.061
Lipid-lowering drugs			
Statins	102 (93.6)	152 (96.1)	0.294
Fibrates	17 (15.5)	32 (20.1)	0.115
Ezetimibe	13 (11.8)	22 (13.8)	0.442
RAAS inhibitors			
ACEI	35 (31.8)	57 (35.8)	0.176
ARB	16 (14.5)	15 (9.43)	0.093
ARNI	37 (33.6)	57 (35.8)	0.154
Diuretics	85 (77.2)	132 (83.0)	0.120
MRA	35 (31.8)	64 (40.2)	0.072

ACEI, angiotensin-converting enzyme inhibitors; ARB, angiotensin receptor blockers; ARNI, angiotensin-neprilysin inhibitors; MRA, mineralocorticoid receptor antagonist.

### 3.4 Correlation analysis and visualization

To further elucidate the relationships between key variables and the occurrence of type I cardiorenal syndrome (CRS), correlation analyses were performed, and the results were visualized through scatter plots with trend lines. According to conventional standards, the observed correlations are classified as moderate (*r* = 0.428–0.565), with all *P*-values < 0.01 indicating statistical significance.

**Figure 2:**
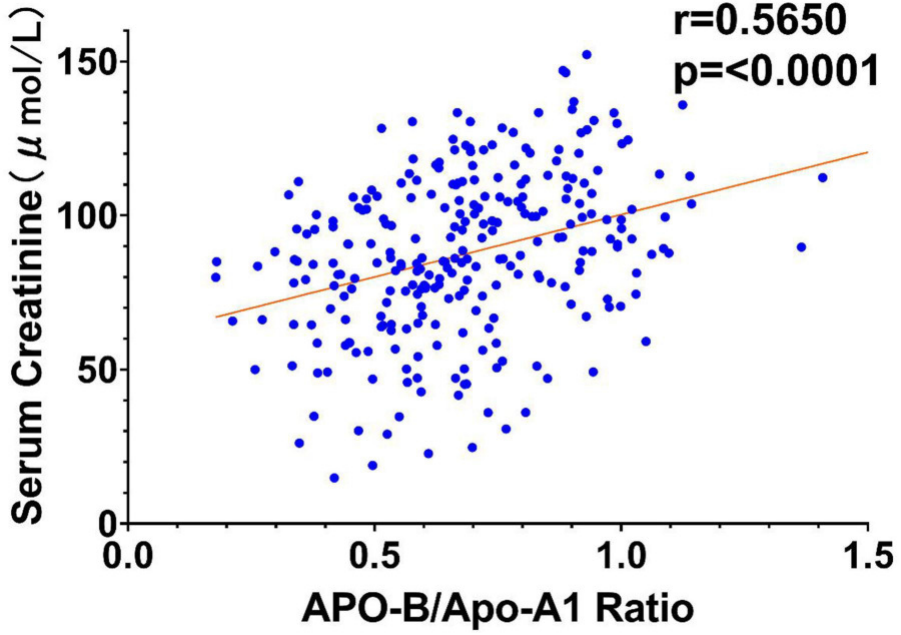
Scatter plot of ApoB/ApoA1 ratio vs. serum creatinine.

Figure 2 presents a scatter plot correlating the ApoB/ApoA1 ratio against serum creatinine concentrations. The data points are distributed along a trend line that slopes upward (*r* = 0.565, *P* < 0.001), indicating a positive moderate association. This relationship underscores the ApoB/ApoA1 ratio as a potential indicator of renal impairment, where elevated ratios correspond with increased creatinine levels, a marker of decreased eGFR.

**Figure 3:**
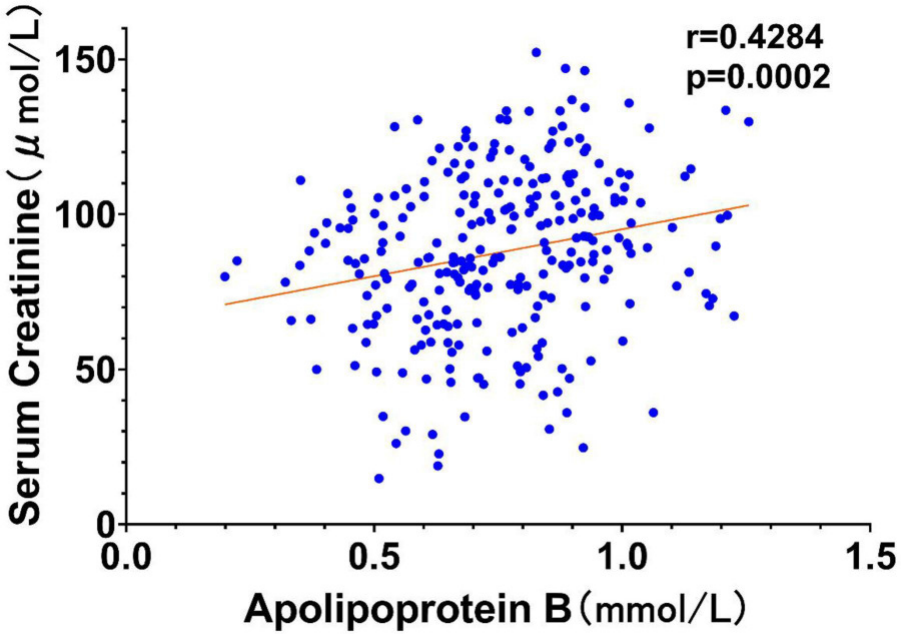
Scatter plot of Apo-B vs. serum creatinine.

In Figure 3, we observe a scatter plot detailing the relationship between serum ApoB concentrations and serum creatinine. The data points align closely with a positive moderate correlation coefficient (*r* = 0.428, *P* = 0.002), suggesting that increased ApoB levels are predictive of higher creatinine levels.

**Figure 4:**
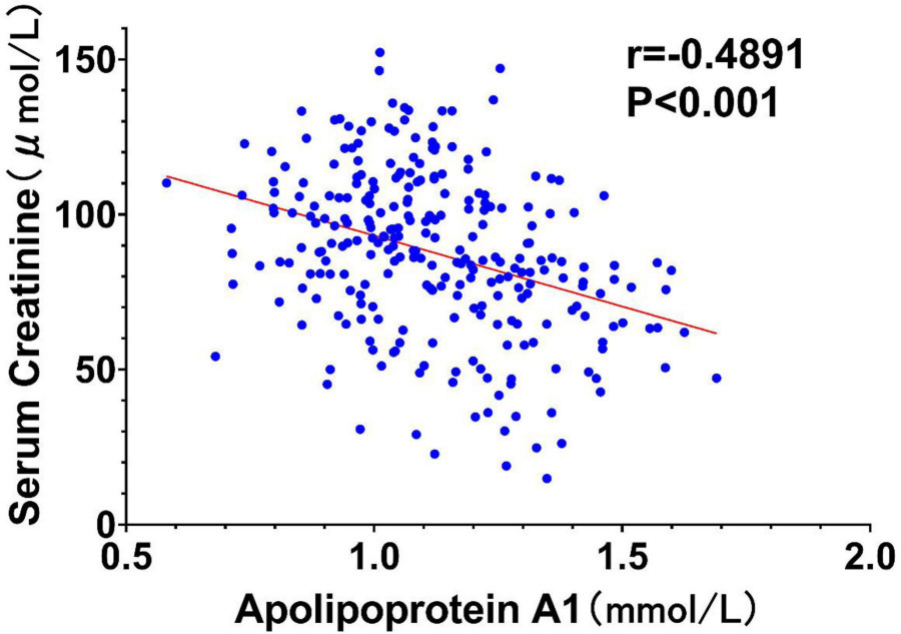
Scatter plot of ApoA1 vs. serum creatinine.

Figure 4 illustrates a contrasting scenario with a scatter plot depicting an inverse moderate correlation between ApoA1 levels and serum creatinine (*r* = −0.4891, *P* < 0.001). The descending trend line indicates that higher ApoA1 levels are associated with lower creatinine levels, suggesting a protective effect against renal decline.

### 3.5 Multivariable logistic regression analysis of cardiorenal syndrome

Multivariable logistic regression analysis was conducted to assess the independent association between the ApoB/ApoA1 ratio and the occurrence of CRS. Covariates included in the model were age, gender, body mass index (BMI), history of diabetes mellitus, history of hypertension, and triglyceride (TG) levels—variables selected based on their clinical relevance to lipid metabolism and cardiorenal function, as well as potential confounding effects.

Table 3 present the findings from a multivariable logistic regression analysis evaluating the predictive factors associated with the development of cardiorenal syndrome (CRS) in patients with coronary artery disease (CAD). The analysis identified several significant predictors of CRS, including age, diabetes mellitus (DM), triglycerides (TG), and the ApoB/ApoA1 ratio. Notably, age (OR = 1.584, 95% CI: 0.955–1.913, *P* = 0.006) and the ApoB/ApoA1 ratio (OR = 1.470, 95% CI: 1.269–1.892, *P* = 0.027) emerged as independent risk factors for CRS.

**Table 3:** Multivariable logistic regression analysis of cardiorenal syndrome (CRS).

Factor	β-value	SE	OR value (95% CI)	*P*-value
Age	0.124	0.017	1.584 (0.955–1.913)	0.006
DM	0.590	0.305	1.164 (0.892–1.380)	0.053
TG	0.441	0.233	1.254 (0.884–1.453)	0.079
ApoB/ ApoA1 Ratio	0.020	0.006	1.470 (1.269–1.892)	0.027

DM, diabetes mellitus; TG, triglycerides.

### 3.6 ROC curve for predicting type I cardiorenal syndrome using the ApoB/ApoA1 ratio

The receiver operating characteristic (ROC) curve was constructed with the ApoB/ApoA1 ratio as the primary variable, incorporating the same covariates to validate the ratio's independent accuracy in identifying Type I CRS.

**Figure 5:**
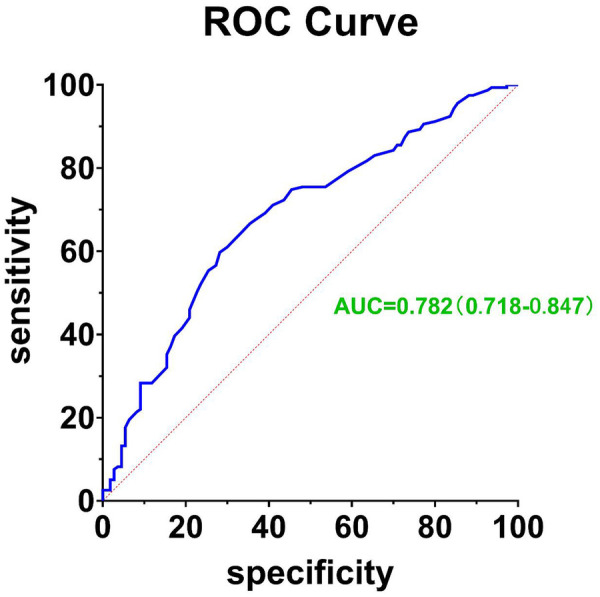
Apob/ApoA1 ratio ROC for type I CRS prediction.

This Figure 5 illustrates the receiver operating characteristic (ROC) curve constructed using the ApoB/ApoA1 ratio as a predictor for Type I cardiorenal syndrome (CRS). The area under the curve (AUC) is 0.782, with a 95% confidence interval ranging from 0.718 to 0.847, indicating a moderate level of predictive accuracy for the model.

## 4 Discussion

Our study provides novel insights into the association between the ApoB/ApoA1 ratio (measured at admission) and the risk of developing Type I CRS (within 7 days of admission) in CAD patients with acute heart failure. The findings indicate that an elevated ApoB/ApoA1 ratio is significantly associated with an increased risk of CRS, aligning with our initial hypothesis.

This study found that the ApoB/ApoA1 ratio of the Type I CRS group was significantly higher than that of the SHF group (0.75 ± 0.21 vs. 0.62 ± 0.19, *P* < 0.001), and multivariate logistic regression analysis confirmed it as an independent risk factor (OR = 1.470, 95% CI: 1.269–1.892, *P* = 0.027), which indicates that the elevated ApoB/ApoA1 ratio is closely related to the occurrence of Type I CRS in CAD patients and is an independent predictive index. The ApoB/ApoA1 ratio, reflecting the balance between atherogenic and anti-atherogenic lipid fractions, may serve as a marker of systemic inflammation and endothelial dysfunction, both of which are implicated in the development of CRS (19, 20). The ApoB/ApoA1 ratio could also indicate a pro-thrombotic state, which is known to contribute to the progression of atherosclerosis and renal impairment (21).

The biological significance of the ApoB/ApoA1 ratio as a predictive factor for cardiorenal syndrome (CRS) is multifaceted. Apolipoprotein B (ApoB) is a major component of low-density lipoprotein (LDL) particles and is closely associated with endothelial dysfunction and inflammation, both of which are key elements in the pathogenesis of atherosclerosis and renal disease. Elevated ApoB levels lead to increased LDL cholesterol, which in turn promotes the formation of atherosclerotic plaques, induces endothelial dysfunction, reduces blood flow, and increases the risk of cardiovascular events. Moreover, a positive correlation exists between ApoB levels and inflammatory markers, indicating a direct link between ApoB and systemic inflammation. Inflammation can further exacerbate endothelial dysfunction and promote renal injury (22, 23).

Conversely, apolipoprotein A1 (ApoA1) is a major component of high-density lipoprotein (HDL) particles and possesses anti-inflammatory and anti-atherosclerotic properties. ApoA1 is involved in reverse cholesterol transport, moving cholesterol from peripheral tissues back to the liver for excretion. This process reduces cholesterol accumulation in arterial walls and lowers the risk of atherosclerosis. Additionally, the anti-inflammatory effects of ApoA1 can protect against endothelial dysfunction and renal damage (24, 25).

The ApoB/ApoA1 ratio reflects the balance between pro-atherogenic and anti-atherogenic lipid fractions. An elevated ratio indicates a shift toward a pro-atherogenic lipid profile, characterized by increased LDL and decreased HDL levels. This imbalance may exacerbate endothelial dysfunction and inflammatory responses, thereby promoting the development of atherosclerosis and renal disease. In the pathophysiology of cardiorenal syndrome (CRS), where dysfunction in one organ (heart or kidney) can exacerbate the other, an elevated ApoB/ApoA1 ratio may serve as a biomarker for increased risk of CRS. It suggests that patients are at a higher risk of cardiovascular and renal complications and are more susceptible to developing CRS (26). In summary, the ApoB/ApoA1 ratio integrates the interplay between pro-atherogenic and anti-atherogenic lipid fractions and is significant for endothelial function, inflammation, and overall cardiovascular and renal health.

The association of age with CRS risk underscores the importance of age as a risk factor in cardiovascular and renal health. The increased risk with age could be attributed to age-related changes in vascular structure and function, as well as cumulative exposure to risk factors over time. Diabetes mellitus was found to be associated with CRS, highlighting the impact of glycemic control on both cardiovascular and renal health (27, 28).

The correlation between the ApoB/ApoA1 ratio and cardiovascular disease (CVD) risk has been the subject of extensive investigation in prior research. Large-scale cardiovascular epidemiological cohorts, including the INTERHEART and AMORIS studies, have both demonstrated that an elevated ApoB/ApoA1 ratio correlates with a higher incidence of cardiovascular events (29, 30). However, research into this specific ratio's association with Type I cardiorenal syndrome remains remarkably limited. In comparison, the present study focuses explicitly on this under-investigated relationship and yields novel exploratory insights into the field.

The strengths of this study are multifold. Firstly, it offers a comprehensive evaluation of the predictive value of the ApoB/ApoA1 ratio for CAD and CRS, which is relatively unexplored. The study's middle sample size of 269 patients provides a solid foundation for robust analysis. Secondly, the data collection was extensive, covering a broad spectrum of clinical characteristics, biochemical measurements, and echocardiographic parameters. This comprehensiveness enhances the reliability of our findings. Lastly, the application of multivariable logistic regression analysis allowed us to adjust for potential confounders, thereby strengthening the validity of our conclusions.

Despite these strengths, our study is not without limitations. A key limitation of this research is its retrospective study design, which inherently introduces inherent biases and may thereby affect the valid causal interpretation of the study outcomes. Additionally, this single-center study means our findings may not be generalizable to other demographic groups or healthcare systems. Selection bias remains a potential issue because the study cohort was drawn from a specific hospital, and thus may not be representative of CAD patients in the general clinical setting. Furthermore, while several confounding factors were considered and adjusted for in our analysis, residual confounding due to unmeasured or unrecorded variables could still exert an influence on the associations we observed.

Moving forward, prospective research is urgently needed to further verify the predictive value of the ApoB/ApoA1 ratio for CRS. For example, it is critical to explore whether the amelioration of lipid profiles achieved with commonly used lipid-lowering agents—including PCSK-9 inhibitors and inclisiran—can mitigate or even reverse the development of cardiorenal syndrome. Multicenter investigations should also be undertaken in future studies to verify the generalizability of our findings across different patient populations and clinical settings. Longitudinal research would help clarify the dynamic temporal association between the ApoB/ApoA1 ratio and the onset and progression of CRS. Moreover, investigating the underlying molecular and pathophysiological mechanisms by which the ApoB/ApoA1 ratio modulates CRS risk may identify novel therapeutic targets. Such investigations could focus on the roles of systemic inflammation, vascular endothelial function, and other relevant pathophysiological processes in the pathogenesis of CRS.

## 5 Conclusion

This study has provided compelling evidence that the ApoB/ApoA1 ratio is moderately associated with the risk of developing Type I cardiorenal syndrome (CRS) in patients with coronary artery disease (CAD). Our findings reveal that patients with CAD who exhibit higher ApoB/ApoA1 ratios are at a greater risk of experiencing CRS, a condition characterized by kidney injury following heart failure exacerbations. This association underscores the potential of the ApoB/ApoA1 ratio as a clinically relevant biomarker that could aid in the early identification of patients at elevated risk for CRS.

The implications of these findings are substantial, as they offer a novel perspective on the pathophysiological mechanisms underlying CRS and suggest a potential target for therapeutic intervention. By identifying patients with CAD who are more likely to develop CRS, clinicians can implement early and targeted interventions aimed at mitigating the progression of this syndrome. This could potentially lead to improved patient outcomes by preventing or delaying the onset of CRS and its associated complications.

In conclusion, our study has not only confirmed the association between the ApoB/ApoA1 ratio and CRS risk but has also highlighted its importance in the clinical management of CAD patients. Future research should focus on validating these findings in prospective studies and exploring the utility of the ApoB/ApoA1 ratio as a predictive tool in clinical practice. This could ultimately lead to more personalized and effective strategies for the prevention and management of CRS in patients with CAD.

## Data Availability

The datasets presented in this study can be found in online repositories. The names of the repository/repositories and accession number(s) can be found in the article/Supplementary Material.
